# Attentional Modulation of Temporal Contrast Sensitivity in Human Vision

**DOI:** 10.1371/journal.pone.0019303

**Published:** 2011-04-25

**Authors:** Isamu Motoyoshi

**Affiliations:** Human and Information Science Lab, NTT Communication Science Labs, NTT, Atsugi, Japan; University of Sydney, Australia

## Abstract

Recent psychophysical studies have shown that attention can alter contrast sensitivities for temporally broadband stimuli such as flashed gratings. The present study examined the effect of attention on the contrast sensitivity for temporally narrowband stimuli with various temporal frequencies. Observers were asked to detect a drifting grating of 0–40 Hz presented gradually in the peripheral visual field with or without a concurrent letter identification task in the fovea. We found that removal of attention by the concurrent task reduced the contrast sensitivity for gratings with low temporal frequencies much more profoundly than for gratings with high temporal frequencies and for flashed gratings. The analysis revealed that the temporal contrast sensitivity function had a more band-pass shape with poor attention. Additional experiments showed that this was also true when the target was presented in various levels of luminance noise. These results suggest that regardless of the presence of external noise, attention extensively modulates visual sensitivity for sustained retinal inputs.

## Introduction

Attention plays a central role for selecting behaviorally relevant information among enormous retinal inputs. The effect of attention has typically been demonstrated as variations in the reaction time for detecting and identifying a clearly visible pattern [1]–[3]. Recently, an increasing number of studies have focused more on the computational mechanisms underlying these attentional facilitations, using simple measures such as detection threshold [4]–[7], discrimination sensitivity [8], [9], and suprathreshold appearance [10]. This approach enables us to analyze the effect of attention more systematically in terms of the gain and selectivity of visual channels and makes it easier to relate the behavior to the attentional modulation of cortical responses [11].

Contrast sensitivity is considered a fundamental behavioral measure of early visual channels [12]. As a primary application of this new paradigm, previous studies have examined whether attention can alter contrast sensitivity. They showed that the contrast sensitivity for a peripheral grating is modulated by bottom-up cueing [6] or by top-down engagement with a concurrent task [4], [5], by an amount of ∼0.15 log unit [5], [6] or less [4]. It has also been shown that the effect is almost independent of the spatial frequency; i. e., attention does not alter the shape of the spatial contrast sensitivity function [6].

The present study investigated the effect of attention on the ‘temporal’ contrast sensitivity function. For decades, temporal contrast sensitivity has also been used as a basic measure of the temporal-frequency characteristics of early visual sensors [13], [14], but the influence of top-down factors has been largely ignored. Here, we used a dual-task paradigm [4], [5] to examine whether the temporal contrast sensitivity for a peripheral drifting grating is altered when observers are attentively engaged in the central letter recognition task. Experiment 1 showed that removal of attention by the concurrent task reduced the contrast sensitivity to a much greater extent for low temporal frequencies than for high temporal frequencies. Experiment 2 showed that this was true even when the target was presented in noise. These results provide evidence that behavioral temporal-contrast sensitivity can be largely altered by top-down attention and support the notion that attention substantially amplifies the gain for sustained image inputs.

## Methods

Experiments were conducted with completed consent forms and permission from the NTT CS Labs Ethical Committee.

### Experiment 1

#### Observers

Seven paid volunteers and the author served as observers. All had normal or corrected-to-normal vision. Paid volunteers were neither researchers nor students and were naive to the purpose of the study.

#### Apparatus

Visual stimuli were displayed on a CRT (SONY GDMF500R) controlled by a graphics card (CRS ViSage). The CRT had a spatial resolution of 1.7 min/pixel at the viewing distance of 1 m we used, and had a refresh rate of 160 Hz. The stimuli were drawn on VRAM as 8-bit gray scale images, and their contrast was controlled by a 14-bit look-up table.

#### Stimuli

**Figure 1:**
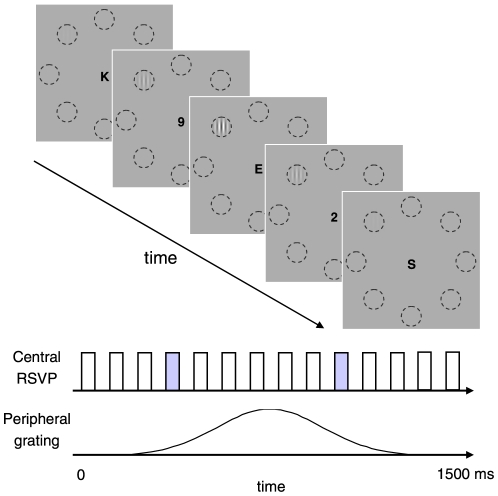
Stimulus sequence in Experiment 1. A vertical grating pattern was presented within a Gaussian temporal window at one of eight locations (denoted by dashed circles, which were not actually shown in the experiment) while small letters were serially presented in the center of the display. The grating was static or drifted at a particular temporal frequency.

All stimuli were presented on a uniform gray background of 68 cd/m^2^ (63 cd/m^2^ for two observers) subtending 23(W)×17(H) deg. The stimulus sequence is illustrated in Fig. 1. The target stimulus was a vertical sinusoidal grating with a spatial frequency of 2.2 c/deg, windowed by a Gaussian with a standard deviation of 0.5 deg (i.e. Gabor patch). The grating was static (0 Hz) or drifted at a particular temporal frequency ranging from 0.63 to 40 Hz. In each trial, the grating was gradually presented within a Gaussian temporal window with a standard deviation of 250 ms, at one of eight possible locations on a virtual circle of 4-deg radius with equal spaces. During the target-presentation period (+−0.75 s around the peak of Gaussian temporal window), a rapid-serial visual presentation (RSVP) display concurrently appeared in the center of the background. We used the RSVP display in order to confine observers' attentional resources throughout the target presentation of 1.5 s [15]–[18]. In our RSVP display, 15 capital alphabetical letters (excluding I, O, Q, Y, Z) were serially presented every 50 ms, separated by a blank of 50 ms (10 Hz). Each letter was drawn in ‘Arial font in black and subtended 0.2×0.2 deg. Two letters were replaced by two numbers randomly chosen from 1 to 9. One of the two numbers appeared at a frame within the 2nd–6th, and the other at a frame within 10th–14th in the sequence, respectively. For five observers who had a difficulty in identifying the numbers, 12 letters were presented every 63 ms with a blank of 63 ms (8 Hz).

For a different set of six observers, the target was a static grating with abrupt onset and offset, which was similar to the ‘flashed’ gratings used in previous studies [4]–[6]. This grating was presented for 250 ms in the middle of the RSVP display.

#### Procedure

There were two separate measurement sessions. In single-task sessions, observers viewed the display with a steady fixation of the central RSVP letters and indicated the target location by a button press (8-alternative forced choice). They were instructed to concentrate on detecting the target while gazing at the central letters. In dual-task sessions, the observers were first asked to identify the two numbers in the central RSVP display. If they identified both in the correct order, they next indicated the location of the peripheral target grating. The observers were strongly encouraged to maintain as high a performance as possible in the central RSVP task. Auditory feedback was given when the observer failed to identify the numbers in the RSVP display. No feedback was given for the detection of the peripheral grating. Since the central RSVP task was difficult, all observers performed several hundred practice trials in the dual-task mode in advance. The data collected in the practice trials were not used in the analysis.

Contrast threshold for the peripheral grating was estimated by means of a staircase procedure. In both task modes, the luminance contrast of the grating was decreased by 0.1 log unit after a correct response and increased by the same amount after an incorrect response. Each staircase corresponded a particular temporal frequency and was randomly interleaved within a session. The contrast thresholds, giving 56.3% correct response (d′∼ = 1.5), were estimated by fitting logistic functions to the proportion correct data using the maximum likelihood method. Each estimate was based on at least 120 trials for single-task sessions and 140 trials for dual-task sessions. For dual-task sessions, only trials in which the observers correctly identified the central numbers were used in the threshold estimation.

### Experiment 2

Previous studies using flashed stimuli have shown that attention plays a major role in the detection of a target among noise [5], [19]. However, the results of Experiment 1 suggested that even without noise, attention has a large impact on detection of a target with low, but not high, temporal frequencies. This makes us wonder how these two factors, noise and temporal frequency, are related in the attentional modulation of target detection. To address this question, we also examined the contrast threshold for static and fast drifting targets embedded in various levels of dynamic noise.

The whole display was filled with a dynamic Gaussian luminance noise consisting of square dots of 7×7 min. The standard deviation (SD) of the noise was varied from 0 to 0.08. The mean luminance of the noise over space and time was 68 cd/m^2^. The images of the noise and target were alternatively displayed at every frame of the CRT (6.25 ms). They appeared to be temporally fused. This was needed in order to independently control the luminance contrast of the target and the noise with the 14-bit look-up table of our graphics card. The noise image was changed to a different one every four CRT frames (50 ms), resulting in a broad spatiotemporal frequency. The central region of the noise was circularly masked by a uniform gray with a diameter of 0.9 deg so that the dynamic noise did not interfere with the RSVP letters. The target grating was static (0 Hz) or drifted at a temporal frequency of 20 Hz. The measurements were separately done for each target temporal frequency (0 and 20 Hz) and for each task mode. Seven observers participated in the measurement for 0 Hz, and six observers for 20 Hz. In each trial, the target grating was presented with the dynamic noise within the same Gaussian temporal window (SD = 250 ms). The noise had a particular SD corresponding to each staircase, which was randomly interleaved. The other conditions were the same as in Experiment 1.

## Results

### Experiment 1

**Figure 2:**
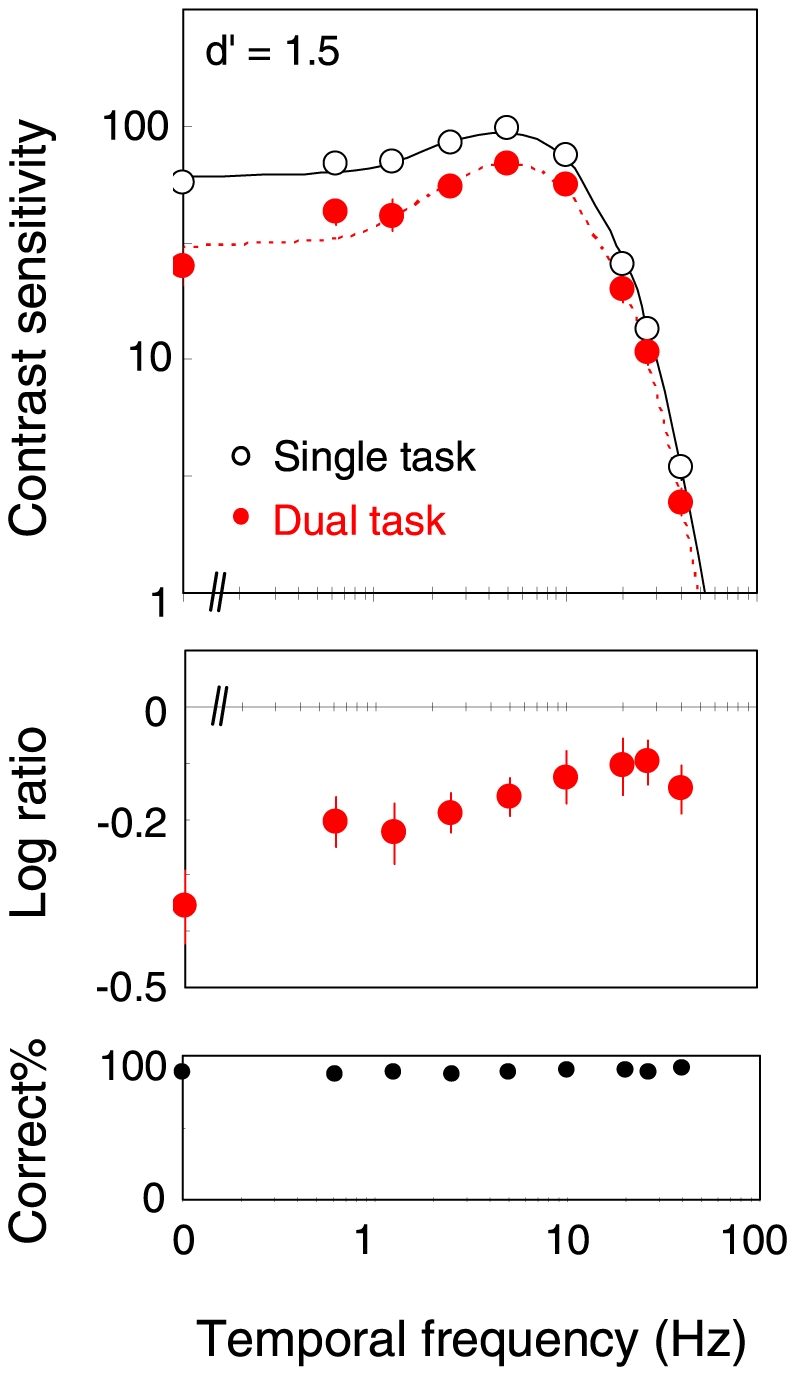
Contrast sensitivity for detecting the peripheral grating at various temporal frequencies. The open circles show the results for the single-task mode, and the red circles the results for the dual-task mode. The middle panel shows the ratio of the sensitivity between the two task modes. The bottom panel shows the proportion correct for the central task. Error bars are +−1 s.e.m. across observers.

The upper panel in Fig. 2 shows the contrast sensitivity as a function of the temporal frequency of the peripheral grating. The data represent the average for the eight observers. The open circles show the results for the single task condition, and the red circles the results for the dual task condition. The smooth curve is the conventional temporal modulation-transfer function (MTF) fitted to the data, which will be described later in more detail.

It is found that the contrast sensitivities in the dual-task mode are lower than in the single-task mode over all temporal frequencies (t-test in log scale; p<0.005 for TFs<5 Hz, and p<0.04 for TFs>5 Hz) except for 20 Hz (p = 0.07). The sensitivity is reduced in the dual-task condition more profoundly at low temporal frequencies. The middle panel shows the ratio in the contrast sensitivity between the two task modes; a lower value indicates a larger sensitivity reduction in the dual-task mode. It is clear that the sensitivity reduction is larger for lower temporal frequencies (one-way ANOVA; F(8, 63) = 2.87, p = 0.009). The sensitivity reduction is only ∼0.1 log unit for high temporal frequencies, while it is ∼0.3 log unit for low temporal frequencies. The contrast sensitivity for the flashed grating was also lower for the dual task than for the single task (t-test in log scale, p = 0.007). The ratio was 0.11 log unit, which is comparable to the data obtained for flashed gratings in previous studies [5], [6]. The bottom panel shows the proportion correct for the central RSVP task, which is within the range of 86.8 to 91.3% across all observers, and shows no dependence upon the target temporal frequency (F(8, 63) = 0.58, p = 0.79).

The relatively smaller effect of attention for high-temporal-frequency stimuli may appear due to the fact that the observers paid attention on the central letters that changed at a high temporal frequency. In additional experiments, we sought to examine this possibility using central displays with relatively low temporal frequencies, such as a gradually presented single letter. However, we found that these displays were not strong enough to confine observers' attentional resource throughout the target presentation, and found no significant effect of the task mode. The present study leaves this “temporal-frequency selective attention” hypothesis for future investigations.

#### System analysis

To analyze quantitatively the change in the temporal contrast sensitivity function between task modes, we fitted the data of each observer by a temporal MTF as follows:
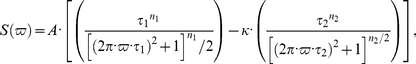
(1)where *τ_1_* and *τ_2_* are the center frequencies, and *n_1_* and *n_2_* the filter widths. The *κ* is a parameter related to the negative phase in the impulse response in the time domain, called the ‘transient factor’ [20]; *κ* = 0 when the MTF is low pass, and κ>0 when it's band pass. The *A* is a scaling factor denoting the overall sensitivity. We fitted this function to the data for individual observers by means of the least-square method on the log scale, assuming identical *τ_1_*, *τ_2_*, *n_1_*, and *n_2_* for both task modes and different *A* and *κ* for each task mode. The fitting was very good for all observers. On average, the RMS error of the fitting was 0.25, and the correlation coefficient between the fitted and observed data was 0.99.

**Figure 3:**
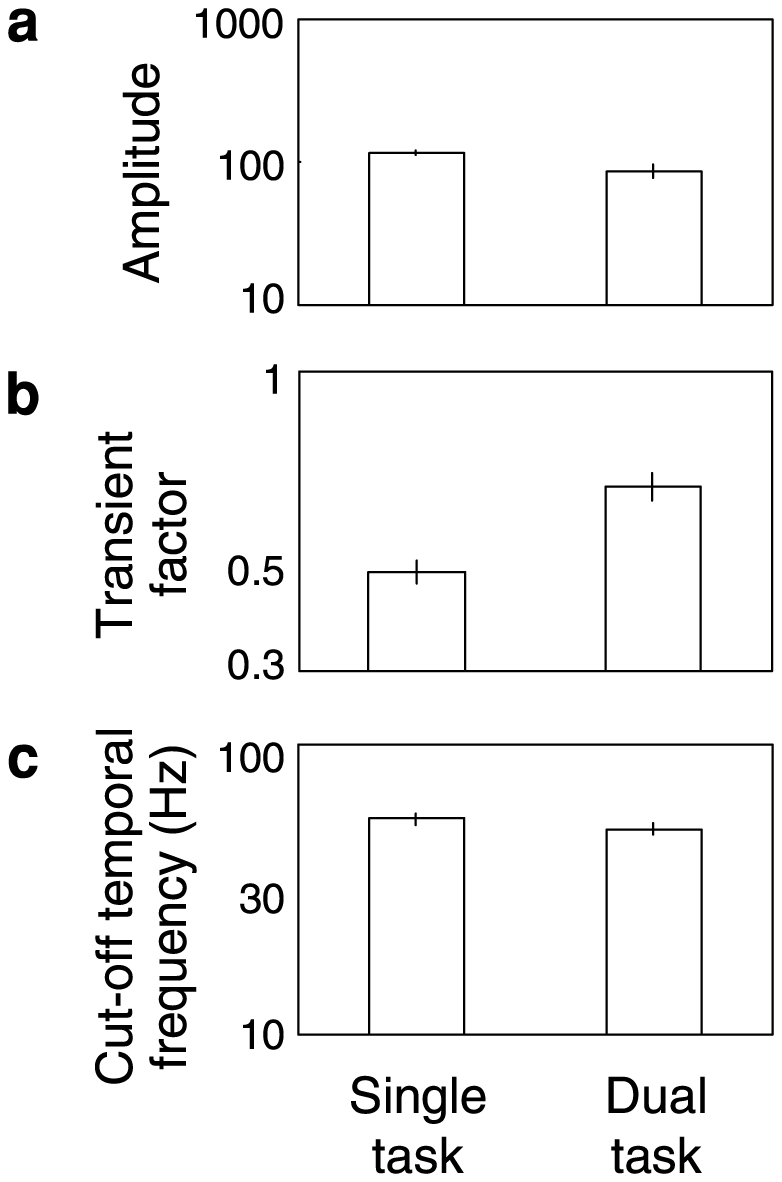
Estimated parameters of the MTF for single- and dual-task conditions. (a) Overall amplitude. (b) Transient factors. (c) Cut-off temporal frequency in hertz. Error bars are +−1 s.e.m. across observers.

The average estimates of *τ_1_*, *τ_2_*, *n_1_*, and *n_2_* were 0.007, 0.02, 5.30, and 19.0, respectively. The overall amplitudes (*A*) were 116.1 for the single task and 89.5 for the dual task. They are shown in Fig. 3a. When compared on the log scale, they were significantly different (t-test, p = 0.01). The transient factors (*κ*) were 0.47 for the single task and 0.65 for the dual task (Fig. 3b), suggesting that the contrast sensitivity function had a more band-passed shape in the dual-task mode (t-test in log scale, p = 0.0008). By extrapolating the fitted curve, we also estimated the intercept of the function with 1.0, which was regarded as the cut-off temporal frequency. As shown in Fig. 3c, the estimates were 55.2 Hz for the single task and 51.2 Hz for the dual task (t-test in log scale, p = 0.03), indicating a slight decrease of the temporal resolution due to the concurrent task.

#### Slope of psychometric functions

**Figure 4:**
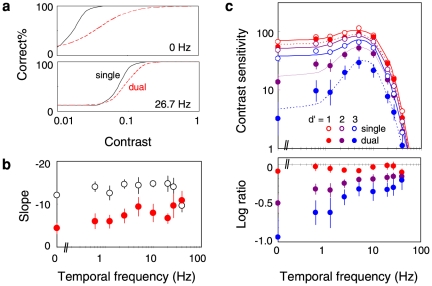
Effect on the slope of psychometric function. (a) Examples of psychometric functions for the peripheral grating detection. The upper panel shows the results for the static grating (0 Hz) and the lower panel the results for the fast drifting grating (26.7 Hz). The solid curves are the psychometric functions for the single-task mode, and the red dashed curves are those for the dual-task mode. (b) Slope of psychometric functions plotted as a function of the grating temporal frequency. The open circles shows the results for the single-task mode, and the red circles the results for the dual-task mode. Error bars are +−1 s.e.m. across observers. (c) Contrast sensitivity defined with different performance criteria; the red symbols for d′ = 1.0, purple symbols for d′ = 2.0, and blue symbols for d′ = 3.0. The lower panel shows the ratio in the sensitivity between the two task modes. Error bars are +−1 s.e.m. across observers.

In addition to the elevation of the threshold, we also found that the slope of the psychometric function tends to be shallower in the dual-task mode. Fig. 4a illustrates two typical psychometric functions for the static gratings (0 Hz, upper panel) and drifting gratings of a high temporal frequency (26.7 Hz, lower panel), both of which were drawn using the averaged threshold and slope across observers. The psychometric function for the static grating appears considerably shallower in the dual task (red dashed curve) than in the single task (black solid curve). Figure 4b plots the averaged slope as a function of the temporal frequency of the grating. Except for very high temporal frequencies (26.7 and 40 Hz, p>0.13), the slope is shallower in the dual-task mode than in the single-task mode (t-test, p<0.004 for TFs less than 1.25 Hz, p<0.05 for TFs from 1.25 to 20 Hz). The decrease in the slope appears a little more profound when the temporal frequency is low, but this trend was not significant (one-way ANOVA, F(8, 63) = 1.74, p = 0.11). The partial-correlation analysis revealed that the decrease in the slope was correlated with the decrease in the sensitivity (r = 0.26, p = 0.03) but not with the temporal frequency (r = 0.19, p = 0.11).

From these results, it is expected that the amount of sensitivity reduction in the dual task would depend on the criteria of the correct detection that defines the threshold. Figure 4c shows the contrast sensitivity function, and the ratio, as defined by the threshold that gives proportion correct of 39% (d′ = 1; red symbols), 71% (d′ = 2; purple symbols), and 92% (d′ = 3; blue symbols). The plots show that the concurrent task little affects the sensitivity defined at low proportion correct (d′ = 1; red symbols) but greatly reduces that defined at high proportion correct (d′ = 3; blue symbols). In the latter case, the sensitivity is reduced as much as ∼10 times (i.e., ∼1.0 log unit) for low temporal frequencies. This is far greater than for flashed gratings (∼0.1 log unit) with the similar criterion (90%) in a previous study [6].

We also analyzed the data assuming a psychometric (logistic) function with a variable asymptote level as well as a variable center and slope. The results showed that changes in the slope across task modes were partially replaced by those in the asymptote level. Although the parameter estimates in this analysis were a little unstable, probably because our staircase procedure collected a small number of data at high contrast, this implies the effect of attention on the asymptote level as also reported in previous studies [7]. Importantly, this analysis also resulted in criteria-dependent sensitivity modulations similar to those found in the original analysis. For 0 Hz target, for example, the sensitivity reduction by the central task was 0.16 log unit at d′ of 1 (p = 0.001) and 0.82 log unit at d′ of 2 (p = 0.016), and that for 20 Hz target was 0.03 log unit at d′ of 1 (p = 0.5) and 0.34 log unit at d′ of 2 (p = 0.11), respectively.

#### Fixational eye movements

The sensitivity reduction in dual tasks might be caused by suppression of fixational eye movements, which can affect the contrast sensitivity for low temporal frequencies [14]. To test this possibility, we measured the observers' fixational eye movement using an eye tracking device (SR Research Ltd. EyeLink II, 500 Hz), although this was done in a separate session because the device was installed at a different place. Eight observers were asked to perform the single and dual tasks as they did in the main experiment. The power spectrum of the horizontal eye position was calculated for trials without a large and rapid eye movement (>200 deg/sec) due to blink or saccade. The results showed that the average power spectrum across observers did not significantly differ between task modes at any temporal frequency from 1 to 100 Hz (t-test in log scale, p>0.13). We also found that the average distance of the eye position from the baseline (initial 200 ms of the trial) was 0.6 (+−0.16, s.e.m.) deg for single tasks and 0.4 (+−0.06) deg for dual tasks (t-test, p = 0.07). This difference (∼0.2 deg) is very small with respect to the target eccentricity of 4 deg. These results are not consistent with the notion that fixational eye movement alone explains variations in the contrast sensitivity between the two task modes.

### Experiment 2

**Figure 5:**
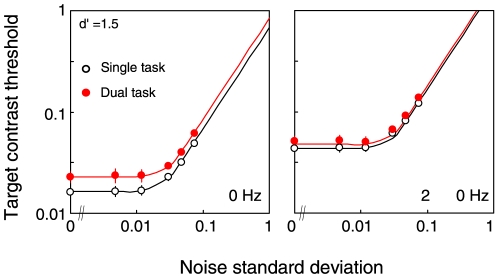
Contrast thresholds for the target grating as a function of the standard deviation of the noise. The open circles show the results for the single-task mode, and the red circles the results for the dual-task mode. Error bars are +−1 s.e.m. across observers. The left panel shows the results for the static grating (0 Hz); the right panel the results for the drifting grating (20 Hz).

Figure 5 shows the contrast threshold for the target grating as a function of the noise standard deviation. The average threshold data across observers are shown. It is found that the threshold is constant until a certain noise level and then it increases. The threshold is higher for the dual-task mode (filled symbols) than for the single-task mode (open symbols) when the target is static (t-test in log scale, p<0.02 except for the noise level of 0.005 and 0.01), but not significantly so when the target is drifting at 20-Hz (t-test in log scale, p>0.05 for all noise levels). A three-way ANOVA showed significant effects of temporal frequency, task mode, and noise level (F(1,11) = 48.0, p<0.0001, F(1,11) = 32.2, p<0.0002, F(5,55) = 274.4, p<0.0001), and an interaction between task mode and temporal frequency (F(1, 11) = 4.85, p = 0.049). These results suggest that the effect of task mode is larger for low temporal frequencies than for high temporal frequency.

#### PTM analysis

Lu & Dosher [21], [22] have introduced a powerful paradigm called the perceptual template model (PTM) to analyze the effect of attention on visual processing in terms of signal detection. We employed the PTM to analyze our noise-versus-threshold functions. The PTM assumes that the target threshold is determined by three hypothetical noise components: additive internal noise, multiplicative internal noise, and external noise. A reduction in the additive internal noise is related (but not equivalent) to the enhancement of the target signal; for more details about the rationale , see Ref. [21]. To estimate how much each noise is varied with attentional control, the threshold data of individual observers were fitted by a formula taken from Dosher & Lu [22] using the least-square method on the log scale:
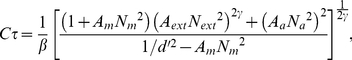
(2)where *Cτ* is the threshold contrast, *γ* the nonlinearity parameter, and *β* the scaling factor. The *d′* is the performance level that defines the threshold. We considered *d′* of 1.0 and 1.5 in the present analysis. *N_a_* indicates the additive noise, and *N_m_* the multiplicative noise. *N_ext_* is the external noise contained in the stimuli. These were assumed to be constant across the task mode. To allow the amount of noise to vary with the task mode, each *N* was multiplied by *A_a_*, *A_m_*, and *A_ext_*, which were assumed to be 1.0 in the dual-task mode but a variable in the single-task mode.

For the data for the 0-Hz target, the average estimates across observers were (*β*, *γ*, *N_a_*, *N_m_*) = (1.59, 2.03, 0.03, 0.18). The three multiplicative factors were estimated as (*A_a_*, *A_m_*, *A_ext_* ) = (0.77, 0.24, 0.83). All of them were significantly lower than 1.0 (t-test, p<0.003), indicating a reduction in all classes of the noise in the single-task mode. The reduction was more profound in the multiplicative noise than in the other two (t-test, p<0.02). This is interpreted as suggesting that for a static target, attention is effective not only for excluding the external noise but also for reducing the internal noise and amplifying the target signal. We also applied the same analysis to the data for the 20-Hz target and found the average estimates of (*β*, *γ*, *N_a_*, *N_m_*, *A_a_*, *A_m_*, *A_ext_*) = (0.63, 2.17, 0.02, 0.01, 0.87, 0.93, 0.90). Only *A_ext_* was significantly lower than 1.0 (t-test, p<0.005), indicating the exclusion of external noise. But the effect was quite subtle (single ∶ dual = 0.90 ∶ 1.00).

To summarize, these results support the notion that regardless of the amount of external noise, attention has a large impact on the detection of sustained, or low-temporal-frequency, stimuli via enhancement of the signal and suppression of the noise, whereas it has little impact on the detection of transient, or high-temporal-frequency, stimuli.

## Discussion

The present results show that when the observers concentrate on a different cognitive task, contrast sensitivity is reduced by a large amount for peripheral gratings of low temporal frequencies but little for those of high temporal frequencies. The results are consistent with the previous findings that attention alters contrast sensitivity [6], [7], and further demonstrates that the effect is particularly profound for low temporal frequencies.

The large decline of contrast sensitivity for low temporal frequencies is also qualitatively consistent with recent psychophysical findings that the lack of focal attention rather enhances temporal discrimination of suprathreshold stimuli [23]. Although our analysis showed that attention increases, rather than decreases, the upper temporal frequency limit *per se.*, the band-passed temporal frequency characteristics obtained in the poorly-attended condition is indeed more suitable for temporal discrimination of dynamic stimuli. These findings may support the hypothesis that attention amplifies the output of parvocellular channels, which are sensitive to low temporal and high spatial frequencies [23] and to stimuli at isoluminance [24]. However, it is not always clear that attention largely modulates the contrast sensitivity for any stimuli preferred by parvocellular channels. For example, it has been shown that attentional effects on the detection of a flashed target are weak across spatial frequencies, including high ones [6]. Our pilot observations also indicated that the concurrent task affected only little the detection (d′ = 2) of a drifting grating (10 Hz) even when it had a high spatial frequency (∼0.2 log unit, N = 9) or when it was red-green isoluminant (∼0.1 log unit, N = 6). The parvo-modulation hypothesis may be further tested by considering many differential aspects between two channels, including the gain and saturation level.

From a functional viewpoint, it is nevertheless possible to consider that attention enhances contrast detection by promoting ‘sustained’ signals, regardless of their physiological correlates. It is well known that transient signals produce an extremely strong saliency to capture one's attention almost unconditionally [25] unless they are interfered by other transients in neighbors [26]. Particularly for the contrast detection on a uniform visual field, it is likely that such transient signals automatically prompt the conscious awareness of the target itself. This might result in the weak attentional modulation of the contrast sensitivity for the high-temporal-frequency target in our data and for the flashed target in previous data [4]–[6]. Attention may be effective for enhancing ‘sustained’ information that was not detected by such transient processes.

The contrast detection threshold is essentially based on the conscious awareness of the target stimulus. Recent psychophysical and imaging studies have demonstrated the unconscious processing of invisible stimuli in situations of masking and adaptation [27]–[30], indicating that the behavioral contrast threshold is determined by multiple levels of neural processes [31]. Interestingly, these invisibilities are frequent for static or sluggish stimuli and rare for transient stimuli [27], [28], supporting the notion that visual awareness is gated by transients [29]. Attentional modulation of the contrast sensitivity for sluggish stimuli may also originate in such gate processes beyond early visual channels. This notion is consistent with physiological findings that attentional modulations are often observed in the later, feedback-related, phase of neural responses [32].

It has been shown that endogenous attentional control has a larger impact on the sensitivity for detecting a target presented with noise or distracters than for detecting one presented alone [5], [22], [33]. These findings are consistent with the idea that top-down attention plays a major role in the suppression of irrelevant inputs; c.f., external-noise exclusion [22] or the winner-take-all network [5]. The present results may afford a different interpretation in terms of the temporal characteristics of stimuli. In previous studies, the target and the noise (or distracters) were both briefly flashed in synchrony [5], [33] or in temporal fusion [22]. In such stimuli, transient signals are ineffective for distinguishing between a target and noise, and the visual system must specifically make use of sustained (non-transient) information, which could be enhanced by attention. In our Experiment 2, the 20-Hz drifting grating is likely distinguished from the dynamic white noise by differences in temporal information. Transient signals are available there, and probably little attention was required to find the target. It is therefore possible that the top-down attention plays a role not only in suppressing noise regardless of the temporal property of stimuli, but also in modulating sustained information regardless of the presence of noise. It should be noted, however, that exogenous attentional cues can have larger attentional effects for targets without noise than for those with noise [21]. It is unclear if the above hypothesis is relevant for the exogenous attentional control, which may drive different attentional mechanisms [34], [35] or directly facilitate the detection of stimuli in spatiotemporal neighbors.

The present data also showed that attentional control alters the slope (and/or asymptote level) of the psychometric function. In accordance with the perceptual template model, the change in the slope is a strong signature of the change in multiplicative internal-noise [21]. The present results can be interpreted as indicating that attention modulates the response gain, as well as the input gain, of sustained visual channels. This appears consistent with the response gain modulation found among cortical neurons [11]. However, it should be noted that the psychometric function is determined by a stochastic process based on many binary responses. For example, fluctuations in the input gain across trials can also result in a shallow slope of the function. Recent studies show that the attentional effect on the slope of the psychometric function depends on many factors in stimuli and observers [36], [37]. Also, the type of physiological gain control is also known to depend on stimulus configurations such as size [38]–[40]. More detailed analyses of the psychometric function may unveil new aspects of attention, such as discrete sampling [41].

The present findings were obtained for threshold detection. It is not very easy to tell what implications they have for the effect of attention on the perception and discrimination of suprathreshold stimuli. Discrimination is determined through more complex processes, and is not a simple function of the activities of underlying units [8], [23]. Nevertheless, it would be interesting to revisit the past suprathreshold data in terms of the temporal characteristics.

## References

[1] Posner M (1986). Chronometric Explorations of Mind..

[2] Treisman AM, Gelade G (1980). A feature-integration theory of attention.. Cognitive Psychology.

[3] Wolfe JM, Cave KR, Franzel SL (1989). Guided search: an alternative to the feature integration model for visual search.. Journal of Experimental Psychology Human Perception and Performance.

[4] Lee DK, Koch C, Braun J (1997). Spatial vision thresholds in the near absence of attention.. Vision Research.

[5] Lee DK, Itti L, Koch C, Braun J (1999). Attention activates winner-take-all competition among visual filters.. Nature Neuroscience.

[6] Carrasco M, Penpeci-Talgar C, Eckstein M (2000). Spatial covert attention increases contrast sensitivity across the CSF: support for signal enhancement.. Vision Research.

[7] Carrasco M (2006). Covert attention increases contrast sensitivity: Psychophysical, neurophysiological and neuroimaging studies.. Progress in Brain Research.

[8] Yeshurun Y, Carrasco M (1998). Attention improves or impairs visual performance by enhancing spatial resolution.. Nature.

[9] Yeshurun Y, Carrasco M (1999). Spatial attention improves performance in spatial resolution tasks.. Vision Research.

[10] Carrasco M, Ling S, Read S (2004). Attention alters appearance.. Nature Neuroscience.

[11] Reynolds JH, Pasternak T, Desimone R (2000). Attention increases sensitivity of V4 neurons.. Neuron.

[12] Campbell FW, Robson JG (1968). Application of Fourier analysis to the visibility of gratings.. Journal of Physiology.

[13] Robson JG (1966). Spatial and temporal contrast sensitivity functions of the visual system.. Journal of the Optical Society of America.

[14] Kelly DH (1979). Motion and vision. II. Stabilized spatio-temporal threshold surface.. Journal of the Optical Society of America.

[15] Reeves A, Sperling G (1986). Attention gating in short-term visual memory.. Psychological Review.

[16] Shapiro KL, Raymond JE, Arnell KM (1994). Attention to visual pattern information produces the attentional blink in rapid serial visual presentation.. Journal of Experimental Psychology Human Perception and Performance.

[17] Tsushima Y, Sasaki Y, Watanabe T (2006). Greater disruption due to failure of inhibitory control on an ambiguous distractor.. Science.

[18] Ariga A, Yokosawa K (2008). Attentional awakening: gradual modulation of temporal attention in rapid serial visual presentation.. Psychological Research.

[19] Dosher BA, Lu ZL (2000). Mechanisms of perceptual attention in precuing of location.. Vision Research.

[20] Watson AB, Boff K, Kaufman L, Thomas JP (1986). Temporal sensitivity.. Handbook of Perception and Human Performance, Vol. 1: Sensory Processes and Perception..

[21] Lu ZL, Dosher BA (1998). External noise distinguishes attention mechanisms.. Vision Research.

[22] Dosher BA, Lu ZL (2000). Noise exclusion in spatial attention.. Psychological Science.

[23] Yeshurun Y, Levy L (2003). Transient spatial attention degrades temporal resolution.. Psychological Science.

[24] Yeshurun Y (2004). Isoluminant stimuli and red background attenuate the effects of transient spatial attention on temporal resolution.. Vision Research.

[25] Yantis S, Jonides J (1990). Abrupt visual onsets and selective attention: voluntary versus automatic allocation.. Journal of Experimental Psychology Human Perception and Performance.

[26] Yantis S, Hillstrom AP (1994). Stimulus-driven attentional capture: evidence from equiluminant visual objects.. Journal of Experimental Psychology Human Perception and Performance.

[27] Breitmeyer B, Ogmen G (2006). Visual Masking: Time Slices Through Conscious And Unconscious Vision.

[28] Bonneh YS, Cooperman A, Sagi D (2001). Motion-induced blindness in normal observers.. Nature.

[29] Motoyoshi I, Hayakawa S (2010). Adaptation-induced blindness to sluggish stimuli.. Journal of Vision.

[30] Blake R, Logothetis NK (2002). Visual competition.. Nature Reviews Neuroscience.

[31] Fang F, He S (2005). Cortical responses to invisible objects in the human dorsal and ventral pathways.. Nature Neuroscience.

[32] Lamme VA, Roelfsema PR (2000). The distinct modes of vision offered by feedforward and recurrent processing.. Trends in Neurosciences.

[33] Joseph JS, Chun MM, Nakayama K (1997). Attentional requirements in a ‘preattentive’ feature search task.. Nature.

[34] Briand KA (1998). Feature Integration and Spatial Attention: More Evidence of a Dissociation Between Endogenous and Exogenous Orienting.. Journal of Experimental Psychology Human Perception and Performance.

[35] Hein E, Rolke B, Ulrich B (2006). Visual attention and temporal discrimination: Differential effects of automatic and voluntary cueing.. Visual Cognition.

[36] Cameron EL, Tai JC, Carrasco M (2002). Covert attention affects the psychometric function of contrast sensitivity.. Vision Research.

[37] Pestilli F, Viera G, Carrasco M (2007). How do attention and adaptation affect contrast sensitivity?. Journal of Vision.

[38] Martinez-Trujillo J, Treue S (2002). Attentional modulation strength in cortical area MT depends on stimulus contrast.. Neuron.

[39] Reynolds JH, Heeger DJ (2009). The normalization model of attention.. Neuron.

[40] Herrmann K, Montaser-Kouhsari L, Carrasco M, Heeger DJ (2010). When size matters: attention affects performance by contrast or response gain.. Nature Neuroscience.

[41] VanRullen R, Carlson T, Cavanagh P (2007). The blinking spotlight of attention.. Proceedings of the National Academy of Sciences of the United States of America.

